# Regulatory considerations to keep pace with innovation in digital health products

**DOI:** 10.1038/s41746-022-00668-9

**Published:** 2022-08-19

**Authors:** John Torous, Ariel D. Stern, Florence T. Bourgeois

**Affiliations:** 1 Beth Israel Deaconess Medical Center, Harvard Medical School, Boston, MA USA; 2 Harvard Business School, Boston, MA USA; 3 Digital Health Center, Hasso Plattner Institute, Potsdam, Germany; 4 Harvard-MIT Center for Regulatory Science, Harvard Medical School, Boston, MA USA; 5 Computational Health Informatics Program (CHIP), Boston Children’s Hospital, Boston, MA USA; 6 Department of Pediatrics, Harvard Medical School, Boston, MA USA

**Keywords:** Translational research, Rehabilitation

## Abstract

Rapid innovation and proliferation of software as a medical device have accelerated the clinical use of digital technologies across a wide array of medical conditions. Current regulatory pathways were developed for traditional (hardware) medical devices and offer a useful structure, but the evolution of digital devices requires concomitant innovation in regulatory approaches to maximize the potential benefits of these emerging technologies. A number of specific adaptations could strengthen current regulatory oversight while promoting ongoing innovation.

The COVID-19 pandemic has accelerated interest in digital health, with an unprecedented rise in investment in and use of digital tools as medical devices to diagnose, treat, and manage a wide array of medical conditions^1^. Looking to fill the need for access to care beyond the capacity of video visits, asynchronous telehealth and largely self-help patient-driven digital solutions are moving from consumer novelties towards regulated therapeutic products. Behavioral health has become an inadvertent leader given the mounting burden of mental illness, insufficient access to care, and the relative ease of offering mental health care services via remote sensors and screens^2^. The rapid uptake of such digital tools points to an area ripe with opportunity to improve and complement existing health care services and reach historically underserved populations. But to fully realize the benefits of novel digital offerings, concomitant innovation in regulatory pathways is necessary.

The FDA currently approves and regulates digital treatments that meet the definition of software as a medical device (SaMD). The International Medical Device Regulators Forum defines SaMD as “software intended to be used for one or more medical purposes that perform these purposes without being part of a hardware medical device”^3^. Applications of SaMDs include diagnosis, mitigation, treatment, and prevention of disease. In the United States, SaMD products are primarily regulated through the traditional approaches used to approve lower-risk medical devices, including the de novo and 510(k) pathways. The de novo process is typically used for novel low-risk medical devices (e.g., Class I and II) and requires “reasonable assurance of safety and effectiveness for the intended use.” If sponsors can show substantial equivalence to one or more already marketed devices (i.e., a “predicate device”), they can submit a 510(k) application, which does not require additional safety or efficacy data. The agency may also exercise “enforcement discretion” for certain devices deemed to pose a low risk to patients or designed to help patients manage their disease without providing specific treatment or treatment. Other products are not regulated by the FDA, such as the vast majority of consumer medical apps, as these products are intended to help individuals maintain general fitness, health, or wellness, and do not meet the definition of a medical device.

Given the novelty of digital tools that meet the definition of SaMD, many will utilize the de novo pathway. reSET^®^, for example, is a prescription product that offers web and app-based support for patients with substance use disorder. It is available only by prescription as a prescription digital therapeutic, or “PDT”, and provides 12 weeks of cognitive behavioral therapy (CBT) as an adjunct to standard outpatient therapy to increase treatment retention and abstinence. The company’s website touts impressive clinical trial results and highlights many of the advantages of this therapeutic, including easy accessibility and customizable treatment sessions. However, a 2020 report by the Institute for Clinical and Economic Review questioned the quality of the trial design and noted that the results for the pre-specified primary outcomes did not demonstrate benefit^4^. The de novo clearance for this therapeutic has since been used as the basis for FDA clearance via the 510(k) pathway for two additional PDTs: reSET-O^®^, an adjunct therapeutic for patients with opioid use disorder approved in 2019, and Somryst^®^, a digital CBT therapy for patients with insomnia, approved in 2020. Neither of these products required clinical trial data as part of the FDA clearance process.

Digital tools that meet the definition of a SaMD, but are considered to pose only a low risk to patients, qualify for enforcement discretion and can be made available without active FDA oversight. Examples include software that supports patients to maintain coping skills, engage in behavioral techniques (e.g., CBT), remember to take medications, or receive alerts if their smartphone’s geolocator detects they are in an environment deemed to be unsafe based on their profile (e.g., an individual with alcohol use disorder in a bar). The digital health company Big Health currently releases its mental health and insomnia apps tested in randomized controlled trials through this regulatory scheme. However, without requirements for safety and efficacy data, other companies may make their products available without (rigorous) premarket testing.

The third category, consumer health and wellness apps, are not regulated by the FDA. It is estimated that in 2020, over 90,000 new digital health apps were released, with the majority focused on “wellness management”^5^. As many apps in this space make claims that may be easily interpreted as medical^6^, understanding which apps are targeting wellness vs. formal medical management may not always be straightforward for clinicians and patients^7^. For example, an app offering CBT could fall outside the FDA’s scope if it aims to *promote or encourage healthy … activities generally related to a healthy lifestyle or wellness*, but the same app could qualify for enforcement discretion if it instead offers to help users *maintain their behavioral coping skills by providing a “Skill of the Day” behavioral technique*^8^. When a similar CBT app is used in yet a different manner, for example, as an FDA-approved PDT, it may be regulated by the de novo or 510(k) pathway, as is the case with reSET^®^. This example illustrates how products with similar functions may potentially qualify for different regulatory pathways, highlighting the importance and challenges of maintaining and enforcing clear distinctions between regulatory categories and their respective regulatory requirements. It also underscores how there is room for interpretation in where categorical boundaries lie.

An important consideration in the regulation of digital products and one that is needed to clearly separate unregulated products from those that have formal regulatory clearance or approval is evidence generation to measure the safety, effectiveness, and potential benefit(s) of these products. Currently, the state of evidence for digital health tools has led leaders in the behavioral health space to conclude that “FDA clearance, which focuses on safety and minimal effectiveness thresholds, does not provide adequate information for decision-makers”^9^. Concerns over the effectiveness of digital products is also evident in a 2022 systematic meta-review of 14 meta-analyses of randomized controlled trials for unregulated behavioral health apps, which found that the strength of evidence diminished as the rigor of the study increased^10^.

Rigorously studying digital interventions will require addressing new considerations in study design, including digital placebo effects, digital literacy, health equity, and the generalizability of studies to account for real-world implementation and use. For example, studies that utilize a control group that does not also use technology often report large effect sizes, likely due to a digital placebo effect^11^ Adding a digital placebo, a version of the same app that is missing the “active ingredient,” is an underutilized approach that may help better identify effective apps^10^. For example, Novartis acquired the rights to an app for schizophrenia that had completed two successful but uncontrolled pilot studies demonstrating initial efficacy for improving symptoms. However, when Novartis tested a modified version of the app in a larger study that included a digital placebo, they found no differences between the study arms^12^. Many digital health apps today are complex multimodal interventions and identifying the active ingredient, let alone an appropriate control group remains challenging. Still, identifying active ingredients and their mechanisms of action is important for designing studies that can evaluate digital products.

A consideration in assessing real-world implementation is actual uptake and engagement with digital tools. Data from market research companies suggest that 95% of users stop using a mental health-related app within two weeks^13^. Given such trends, it is unsurprising that some companies have been reticent to share their engagement data. The 2017 introduction of the NASSS (non-adoption, abandonment, scale-up, spread, sustainability) framework^14^ underscores that these challenges are not new. While most drugs and devices approved by the FDA are well tested for the human body or clinical use setting, the variety of potential user environments and myriad use cases for digital tools is far greater. Regulators could consider the implementation and environmental factors that must be in place for optimal effectiveness, such as staff training or access to modern smartphones. This information would help clinicians, patients, and policymakers best evaluate if a product will be useful for specific cases.

To increase real-world evidence for SaMDs, in 2019, the FDA introduced the Digital Health Software Precertification (Pre-Cert) Pilot Program, which aims to evaluate software in a more responsive manner through a focus on developer excellence, review pathway determination, streamlined review, and real-world performance. In 2022, the FDA reports the program is still in its pilot phase and now undergoing simulation testing under different scenarios. While aspects of real-world performance are embedded in Pre-Cert, specific details around these metrics remain to be defined. These details are important considerations; when members of our authorship team attempted to simulate use of the Pre-Cert Program, we found it challenging as apps were not easily matched to simple risk or clinical use functions^15^. Going forward, steps to ensure transparency in the implementation of Pre-Cert, such as standard definitions for user metrics or a public database of app engagement rates and adverse effects, would be useful.

While the potential of harnessing real-world evidence to assess the effectiveness of digital prodcuts is often discussed, there are numerous challenges to consider, including missing data and the validity of digital endpoints^16,17^. For example, when evaluating an app designed to improve mobility after surgery, what should be done with time periods during which no movement data are collected by sensors, not because of the recovery process or because the patient was at rest, but because the device was turned off, was malfunctioning, or the user was not carrying it? Even for less technical approaches that do not involve sensors, there are many cases where simple assessment tools, such as patient-reported outcomes, are reported differently when the same data are collected digitally vs. in person. For example, patient-reported scores for depression and anxiety are not always the same when assessed in different formats as some people may report more severe symptoms to an app vs in person^18^. Relatedly, standards for defining digital biomarkers are still under development (e.g., what constitutes a “step” from a step counter) and determining the validity of digital endpoints remains challenging. Given that smartphones can generate millions of data points per person per day, using clearly defined digital biomarkers is critical to ensure the efficacy of different tools can be compared. While no standard will be perfect, moving forward with one established definition would at least enable real-world data to be better utilized in the evaluation of digital health tools today^16^.

Increasing the use of real-world data from digital tools must also be considered in light of evolving risks beyond current regulatory applications. In the United States, privacy policies for many digital products are insufficient to support the systematic collection of digital data that would be required to implement a robust infrastructure for real-world evidence generation. In February 2022, Crisis Text Line, which offers suicide prevention services, came under national criticism when it was revealed that its privacy policy enabled the transfer of all text message data to a for-profit company selling insights from that data. While Crisis Text Line is not a digital tool, it offers an example of the types of privacy issues plaguing digital health products more broadly. Sharing text message data is not illegal under current regulations and at risk of misuse in the digital health space. A 2021 review of 20,911 apps in the medical and health and fitness categories in the Google play store found that 28% offered no privacy policies and 88% included code that could potentially collect user data^19^. One notable example of a privacy violation occurred with a popular fertility tracking app—one of the most downloaded in 2019—that shared user data, including information on menstruation and pregnancy, with third-party marketing and analytics companies, despite manufacturer assurances that data would only be used internally to support app services. These activities prompted the Federal Trade Commission (FTC) to intervene in January of 2021, ordering the company to notify users of this privacy breach and to obtain users’ explicit consent for any future data disclosures^20^. More robust safeguards will need to be developed along with measures for enforcing compliance before real-world data can be fully leveraged for post-marketing and regulatory use.

While new approaches to ensuring rigorous and transparent regulation of digital health tools will increase public trust and clinical acceptance, there remains the concomitant challenge of ensuring that apps falling outside of regulation are not harmful. In the United States, this will require efforts beyond the FDA, which are beginning to emerge. For example, in 2021 the FTC released a policy statement that digital health apps must follow certain regulations related to notifying consumers about data breaches^20^. Growing concerns about privacy risks and false claims from apps in the wellness space have galvanized interest in new legislation aiming to bring more protection and additional transparency to this currently unregulated space. While outside the FDA’s current scope, addressing privacy risks and degradation of trust in the digital health space may be necessary to ensure the FDA’s vision for the development and regulation of safe and effective digital tools is realized. New solutions like provider organization-specific digital formularies could help patients and clinicians focus on safe and validated digital tools^21^, with some large healthcare organizations already embracing the concept and creating their own ‘digital ecosystems’, such as Kaiser Permanete^22^ and the United States Veteran’s Adminstration^23^. These digital formularies appear highly utilized^22,23^, even though they focus on apps that fall beyond the scope of active FDA regulation.

## Conclusion

Digital health tools are rapidly expanding in scope and adoption, but patients and clinicians struggle to select digital health tools in an environment with inconsistent regulation and sparse information on evidence and uptake. It is encouraging that US policymakers at the FDA are already defining standards and best practices, but more work is needed, including further clarity on regulatory standards coupled with greater transparency around unregulated digital health tools. As a first step, patients and clinicians should be able to readily understand if a product is regulated—and for those products that are regulated, it should be clear that they have been held to an appropriate evidentiary standard in the process. Further, patient-centered controls, such as the types of additional privacy and security requirements that have been put in place for Germany’s regulated digital health apps may be appropriate for the types of SaMD tools that collect and store sensitive health data^16^. A rigorous and well-defined framework for regulating these novel digital tools promises to encourage patient-centered innovation and to catalyze manufacturers to conduct high-quality research in support of safe, effective, and evidence-based products.
